# Identification of zona pellucida defects revealed a novel loss-of-function mutation in *ZP2* in humans and rats

**DOI:** 10.3389/fendo.2023.1169378

**Published:** 2023-05-24

**Authors:** Jun Zeng, Ying Sun, Jing Zhang, Xiaozhu Wu, Yan Wang, Ruping Quan, Wanjuan Song, Dan Guo, Shengran Wang, Jianlin Chen, Hongmei Xiao, Hua-Lin Huang

**Affiliations:** ^1^ Reproductive Medicine Center, Department of Obstetrics and Gynecology, The Second Xiangya Hospital, Central South University, Changsha, Hunan, China; ^2^ Institute of Reproductive & Stem Cell Engineering, School of Basic Medical Science, Central South University, Changsha, Hunan, China; ^3^ Center of Reproductive Health, School of Basic Medical Science, Central South University, Changsha, Hunan, China

**Keywords:** *ZP2*, female infertility, rat model, single-cell RNA sequencing, TGF-β signaling pathway

## Abstract

**Introduction:**

Human zona pellucida (ZP) plays an important role in reproductive process. Several rare mutations in the encoding genes (*ZP1*, *ZP2*, and *ZP3*) have been demonstrated to cause women infertility. Mutations in *ZP2* have been reported to cause ZP defects or empty follicle syndrome. We aimed to identify pathogenic variants in an infertile woman with a thin zona pellucida (ZP) phenotype and investigated the effect of ZP defects on oocyte gene transcription.

**Methods:**

We performed whole-exome sequencing and Sanger sequencing of genes were performed for infertilite patients characterized by fertilization failure in routine *in vitro* fertilization (IVF). Immunofluorescence (IF) and intracytoplasmic sperm injection (ICSI) were used in the mutant oocytes. Single-cell RNA sequencing was used to investigate transcriptomes of the gene-edited (*Zp2^mut/mut^*) rat model. Biological function enrichment analysis, quantitative real-time PCR (qRT-PCR), and IF were performed.

**Results:**

We identified a novel homozygous nonsense mutation of *ZP2* (c.1924C > T, p.Arg642X) in a patient with non-consanguineous married parents. All oocytes showed a thin or no ZP under a light microscope and were fertilized after ICSI. The patient successfully conceived by receiving the only two embryos that developed to the blastocyst stage. The immunofluorescence staining showed an apparently abnormal form of the stopped oocytes. We further demonstrated a total of 374 differentially expressed genes (DEGs) in the transcriptome profiles of *Zp2^mut/mut^* rats oocytes and highlighted the signal communication between oocytes and granulosa cells. The pathway enrichment results of DEGs showed that they were enriched in multiple signaling pathways, especially the transforming growth factor-β (TGF-β) signaling pathway in oocyte development. qRT-PCR, IF, and phosphorylation analysis showed significantly downregulated expressions of Acvr2b, Smad2, p38MAPK, and Bcl2 and increased cleaved-caspase 3 protein expression.

**Discussion:**

Our findings expanded the known mutational spectrum of ZP2 associated with thin ZP and natural fertilization failure. Disruption of the integrity of the ZP impaired the TGF-β signaling pathway between oocytes and surrounding granulosa cells, leading to increased apoptosis and decreased developmental potential of oocytes.

## Introduction

All mammalian eggs are surrounded by a relatively thick extracellular coat, the zona pellucida (ZP), which plays vital roles during the organization and differentiation of granulosa cells, follicle formation, recognition and combination of sperms, stimulation of acrosomal reaction, suppression of polyspermism, and protection of pretransplant embryo (1, 2). Generally, the ZP is composed of three (mice) or four (humans and rats) ZP glycoproteins (ZPGs), varying across different mammalian species (3, 4). Mutations in the ZPG genes have been reported to cause ZP defects and deteriorate egg quality, which are associated with primary infertility in women. Homozygous or compound heterozygous mutations of *ZP1* result in a lack of ZP (5) or empty follicle syndrome (EFS), which is also caused by heterozygous mutations in *ZP3* (6). Biallelic *ZP2* mutations lead to truncated or loss of *ZP2* expression, which produces mature oocytes with a thin, defective ZP or without ZP (7–11). Additionally, a heterozygous variant of *ZP2* has been reported to be a novel cause of EFS recently (12).

Disruption of the integrity of the ZP could decrease the density of the transzonal projections (TZPs) and deteriorate the quality of eggs (13). TZPs are filamentous structures containing elongated cytoplasmic tips that span the ZP to connect the oocytes and surrounding granulosa cells, thereby mediating bidirectional signaling that is crucial for the survival and development of oocytes and granulosa cells. Granulosa cells produce paracrine factors regulating follicular development (14); these factors include the transforming growth factor β (TGF-β) superfamily, comprising TGF-β, Smad2/3, MAPK, activin A, GDF8, and bone morphogenetic protein (BMP) (15–17). Development of oocytes can also be regulated by the extracellular vesicles at the tips of TZPs, which can mediate the intercellular transfer of macromolecules such as mRNAs and miRNAs. Thus, the ZP promotes follicular maturation by maintaining the signal communication between oocytes and granulosa cells.

In the present study, we identified a homozygous truncated mutation in *ZP2* (NM_003460.2, c.1924C > T, p.Arg642X). To investigate the effect of the disruption of ZP integrity on egg development, we used the CRISPR/Cas9 system to create a gene-edited (*Zp2^mut/mut^*) rat model. The advent of single-cell RNA sequencing (scRNA-seq) technology has enabled the detection of changes in transcriptome expression in single cells. Hence, we further used scRNA-seq to determine the molecular pathogenesis during oocyte maturation.

## Materials and methods

### Human subjects

Patients and their family members were recruited from the Reproductive Medicine Center in the Obstetrics and Gynecology Department of the Second Xiangya Hospital, China. Blood samples and immature and/or mature oocytes that remained unfertilized after insemination were used after written informed consent was received from the subjects and donor couples. Human studies were approved by the Ethical Review Board of the Second Xiangya Hospital, Central South University, China.

### Whole-exome sequencing and variant analysis

Genomic DNA was extracted from the peripheral blood samples of the patients and their family members by using the QIAamp^®^ Blood DNA Mini Kit. Exome capture and sequencing were performed using the Agilent SureSelect Whole Exome Enrichment Kit and the Illumina HiSeq 3000 platform. The dbSNP, 1000 Genomes Project, ExAC, and HGMD databases were used to filter the data, and the PolyPhen-2, SIFT, MutationTaster, and CADD were used for functional prediction. Candidate variants were considered based on the following criteria: (i) allele frequency below 1% in any public database, including the gnomAD, ExAC Browser, and 1000 Genomes Project; (ii) nonsynonymous or splice-site variants, or coding INDELs; and (iii) in silico prediction to be pathogenic. Additionally, homozygosity mapping was performed using AutoMap (18), and homozygous variants located in homozygous regions greater than 90 Mb were considered with priority. Candidate variants were subsequently confirmed by Sanger sequencing, and primer sequences were as follows: F′ GTCACCAGGGACAGGTTAAAT and R′ CAAGAGCTCTGGGCAGTAAATA.

### Animals

*Zp2^mut/mut^* rats were generated by CRISPR-Cas9 technology-mediated DNA editing, as reported previously by our team (19). In this study, sexually mature rats (age: 8 weeks) were used. The rats were housed in a controlled environment with a temperature of 23°C and a 12/12h light/dark cycle. Animal protocols were approved by The Animal Care and Use Committee, Central South University, China.

### Oocyte preparation

Oocyte samples for scRNA-seq were prepared using the superovulation technique. We randomly selected one female rat from each group. After administering serum gonadotropin (PMSG, 40IU) from a pregnant mare and human chorionic gonadotropin (HCG, 40IU), the oocytes were ready to be collected. Then, the two female rats were sacrificed by neck removal. The bilateral uterus, ovaries, and fallopian tubes were excised by the abdominal section, and oocytes were isolated under a dissecting microscope. Each cumulus–oocyte complex (COC) was transferred to 0.05% hyaluronidase drop for degranulation. After granulosa cell removal, each oocyte was collected in the polymerase chain reaction (PCR) tube. We collected four oocytes (ZP-intact) from *Zp2^wt/wt^* rat and four oocytes (ZP-absent) from *Zp2^mut/mut^* rat, which were used for the subsequent scRNA-seq (**Supplementary Figure 1**).

### Library construction and scRNA-seq

SMART-seq2 was performed for cDNA synthesis and amplification from cell lysate. The reverse transcription reaction was performed by incubation at 42°C for 90 min, followed by 10 cycles of 50°C for 2 min and 42°C for 2 min, and finally incubation at 70°C for 15 min for inactivation. Before library preparation, the cDNA quantity and quality were assessed by performing agarose gel electrophoresis. The PCR product was thermally denatured into a single strand, and then, the single-strand DNA was cycled with segment-bridge primer to obtain a single-strand circular DNA library. Sequencing libraries were generated according to the manufacturer’s protocol. Then, these libraries were sequenced on the DNBSEQ-500 platform.

### Quality control, data filtering, and normalization

After sequencing, quality analysis was performed on the original data to obtain clean reads. Clean reads were compared with the reference gene sequence using Bowtie2. Hierarchical Indexing for Spliced Alignment of Transcripts (HISAT) was used to compare reference genes. First, HISAT anchored the location of a partial sequence in each read on the genome with a global FM index. Then, the local indexes of these alignment locations were used to align the remaining sequences of each read to extend the alignment area. After the alignment was completed, the reads distributed on the reference sequence were counted.

### Gene clustering analysis

To explore the clusters in different oocytes, we performed principal component analysis (PCA) and visualized the dot plots by selecting the top two principal components.

### Differentially expressed genes and functional enrichment analysis

Genes with at least a two-fold difference and an adjusted *P* value of ≤ 0.05 were identified as DEGs. We used “heatmap” packages to obtain DEG heatmaps. Then, the clusterProfiler software package was used to analyze the selected DEGs. Gene Ontology (GO) enrichment analysis was performed for all the DEGs using DAVID. An adjusted *P* value ≤ 0.05 was regarded as an indication of significant gene enrichment. Furthermore, the Kyoto Encyclopedia of Genes and Genomes (KEGG) enrichment analysis of the DEGs was performed. An adjusted *P* value ≤ 0.05 indicated significant gene enrichment in pathways.

### Quantitative reverse transcriptase PCR

cDNA in *Zp2^wt/wt^* (ZP-intact) and *Zp2^mut/mut^* oocytes (ZP-absent) was analyzed by qRT-PCR to reveal the expression of randomly selected genes (*Esrrg*, *Ehbp1*, *TP53* and *Eno1*) and those in the TGF-β signaling pathway (*Acvr2b* and *Bcl2*). Primers used for qRT-PCR are listed in **Supplementary Table 1**. We used 3–5 eggs for qPCR, following the manufacturer’s protocol of Fast Advanced Cells-to-CT™ qPCR Kit (A35379, Thermo Fisher). Gene expression was normalized against that of the housekeeping gene *Actb* by using the ^ΔΔ^Ct method. Further, fold change was calculated by dividing the normalized gene expression in the *Zp2^mut/mut^* group by the normalized gene expression in the *Zp2^wt/wt^* group.

### Immunofluorescence of ovulated eggs and thawed embryos

Oocytes and embryos for immunofluorescence were collected and fixed in 2% paraformaldehyde for 30 min at 4°C. Then, they were transferred to 0.5% Triton X-100 in phosphate-buffered saline (PBS) for 10 min. After permeabilization, the oocytes were washed thrice with 0.1% Tween-20 in 1 × PBS, 5 min each. Further, the oocytes and embryos were placed in goat serum albumin for 30 min. The oocytes and embryos were incubated with primary antibodies (ZP2: sc-32752, santa-cruz; ZP3: PA5-89033, Thermo Fisher; Acvr2b: sc-376593, santa-cruz; Bcl2: sc-7382, santa-cruz) overnight at 4°C. Then, they were washed with PBS, followed by conjugation with secondary antibodies (GAR0072, GAM0072, lianke) for 2 h at room temperature. Finally, the oocytes and embryos were then stained with DAPI and mounted.

### Ovarian section and histology

Female rats were killed by the intraperitoneal injection of pentobarbital sodium (200 mg/kg body weight), and their ovaries were isolated directly without hormone stimulation. Ovaries were fixed in 4% paraformaldehyde for 2 h, transferred to 70% ethanol, embedded in paraffin, and serially sectioned (5-μm thick); every 10^th^ section was mounted on slides. The slides were deparaffinized using xylene and rehydrated with subsequent ethanol washes.

For immunofluorescence histochemistry, antigen retrieval was performed by microwaving the sections for 7 min in sodium citrate buffer (1 M, pH 6.1). The sections were then placed in goat serum albumin for 20 min at room temperature. They were incubated with the primary antibody (p-Smad2: abs139889, absin; p38MAPK: sc-166182, santa-cruz) overnight at 4°C. After washing with 1 × PBS, the sections were incubated with appropriate secondary antibodies (GAR0072, GAM0072, lianke) for 2 h. Finally, the sections were stained with DAPI to visualize the cell nucleus.

For immunohistochemistry, antigen retrieval was performed using the microwave heating method as follows: ovarian tissue sections were put into 1 L of a boiled sodium citrate antigen retrieval solution; the solution was again boiled for 4 min and then rested for 6 min; the step was repeated three times, and finally, the solution was cooled down naturally. After incubation with a primary antibody (cleaved-caspase3: Asp175, CST) at a dilution of 1:200 overnight, the tissue sections were washed five times with PBS and then were incubated with a secondary antibody (GAM0072, lianke) at 37°C for 1 h. Subsequently, the sections were thoroughly washed and stained with hematoxylin.

### Statistical analyses

Oocyte fluorescence intensity was calculated using the non-commercial image processing package, Fiji-Image J. Five fluorescence images were analyzed for each group. The mean fluorescence intensity in the two groups was calculated. Each experiment was repeated 3 times for statistical analysis. Differences between the groups were analyzed by Chi-square test. All tests were performed using SPSS 19.0 software. P < 0.05 was considered statistically significant.

## Results

### Recruitment of an infertile patient manifesting abnormal *ZP2* phenotype

**Figure 1:**
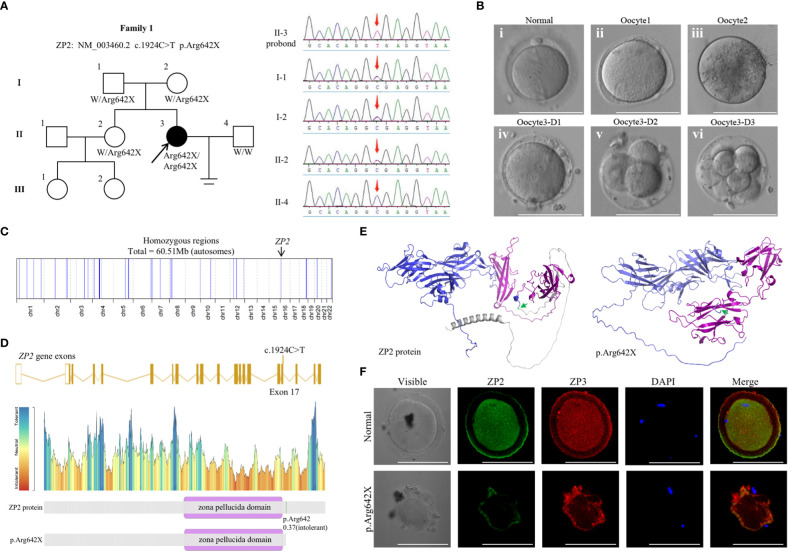
Genotypic and phenotypic features of the patient. **(A)** Identification of the *ZP2* variant. Pedigrees of the patient (family 1) with inherited *ZP2* pathogenic variants (left). Sanger sequencing chromatograms are shown in the right panel and red arrows indicate the *ZP2* mutation. **(B)** Phenotype of the oocytes from the proband (II-3). Three oocytes from the proband were photographed under a light microscope, and a normal MII oocyte (i) is shown for comparison. The oocyte1 (ii) and oocyte3 (iv) presented with a thin ZP, while oocyte2 (iii) presented absence of ZP. The bottom (iv-vi) each represented the morphology of oocyte3 1, 2 and 3 day after fertilized with ICSI. **(C)** Homozygosity mapping of the proband. **(D)** Location of the variant in ZP2. The position of the variant is indicated in the gene structure and protein structure of ZP2. **(E)** The structure prediction of wild-type (left) and mutant image (right) created by Alphafold2 program. The mutation (c.1924C>T p.Arg642X, green arrow) led to protein structure changes as truncation of the protein (gray). Zona pellucida domain was showed in purple. **(F)** Immunofluorescence staining of ZP2 and ZP3 in oocytes of the proband and the control. Green, ZP2; red, ZP3; blue, DAPI. Each treatment was repeated 3 times.

In this study, the proband (II-3) was a 30-year-old woman with 7-year history of primary infertility (**Table 1**; **Figure 1A**). The patient had attained menarche at 15 years of age, and her menstrual cycle, ovarian reserves, and basal sex hormone levels were generally normal (**Supplemental Table 2**). An ultrasonographic scan did not show any abnormalities in the uterus, ovaries, or fallopian tubes, and the chromosomal analysis revealed that the karyotype of the patient was 46, XX. The semen examination of the patient’s husband also showed no obvious abnormality. The pathophysiologic mechanism underlying the infertility could not be identified through routine infertility-related examinations. Considering this unexplained infertility, assisted reproductive technology (ART) treatment was offered to the patient (**Table 1**).

**Table 1:** ART data of the patient.

Cycle number	No. of oocytes retrieved	Fertilization methods	No. of fertilizedoocytes	Stages of oocytes or embryos after fertilization	No. of embryos transferred
1	8	IVF	0	2 in MI, 2 in MII	0
		ICSI	2	1-6C-II, 2-7C-II	2
2	12	ICSI	8	1-8C-II, 2,3-3BC*2, others not formed blastocysts	2

In the first attempt, a gonadotropin-releasing hormone agonist (GnRH-a) protocol was performed, and 8 COCs were retrieved. Considering her long infertile history, 4 COCs were fertilized by ICSI, but 2 were empty after cumulus cell removal; 4 COCs fertilized with IVF remained unfertilized. The 2 embryos formed with ICSI, however, failed to implant after the transfer. The second ICSI attempt was performed according to a progestin-primed ovarian stimulation protocol. A total of 12 COCs were retrieved, including 8 metaphase II oocytes, 2 metaphase I oocytes, one immature oocyte, and one abnormal oocyte. All the oocytes exhibited a relatively thin or no ZP and were fertilized by ICSI and finally formed three frozen embryos (**Figure 1B**). The two blastocysts were thawed successfully and developed into blastocysts, which were then implanted into the patient, leading to successful uterine pregnancy in the next frozen embryo transfer cycle. The proband finally delivered a healthy baby at term successfully and fortunately.

### Whole-exome-identified novel loss-of-function mutation in *ZP2*

Whole-exome sequencing was performed, and a homozygous nonsense mutation in *ZP2* (NM_003460.2 c.1924C>T p.Arg642X) was identified according to the aforementioned strict filtering criteria. Sanger sequencing on DNA from all available members indicated a recessive inheritance pattern (**Figure 1A**). Homozygosity mapping of the proband revealed a large homozygous region with a size of 60.51 Mb (**Figure 1C**). Moreover, the corresponding variant was located in exon 17 and was predicted to result in a premature termination codon (**Figures 1D, E**). Immunofluorescence staining was performed using the frozen-thawed oocytes of the proband and normal controls, which indicated that the ZP of the variant oocytes was fused with the cytomembrane and could not maintain the normal form (**Figure 1F**). These observations indicated an apparently detrimental effect of the variant.

### *Zp2^mut/mut^* rat model generation and transcriptome profile demonstration

**Figure 2:**
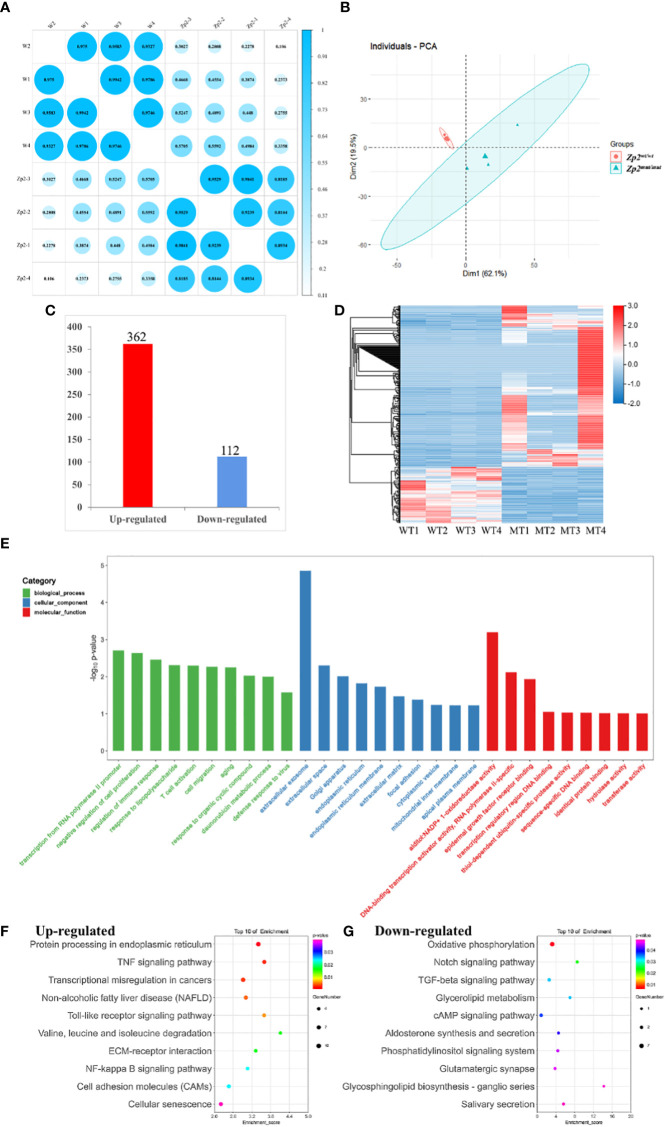
Transcriptional analysis of oocytes from *Zp2^wt/wt^* and *Zp2^mut/mut^* rats. **(A)** Repeated relevance assessment of the transcriptomes of oocytes. **(B)** Principal component analysis (PCA) of eight samples based on gene expression. The red dots indicate *Zp2^mut/mut^* oocytes, and the blue triangles indicate *Zp2^wt/wt^* oocytes. **(C)** Bar chart showing the up-regulated DEGs and down-regulated DEGs. **(D)** Heatmap of all DEGs. Rows represent genes, and columns represent samples. **(E)** GO enrichment of DEGs with the top 10 terms in biological process, cellular component, and molecular function. Color represented the p-value adjust of terms. F-G KEGG enrichment of the top 20 up-regulated **(F)** and down-regulated terms **(G)**. Color represented the p-value adjust of terms.

To further investigate the differences in transcriptome profiles with mutations, we generated homozygous KI rats carrying the loss-of-function *Zp2^mut/mut^* mutation (**Supplementary Figure 1**). Oocyte preparation and scRNA-seq were performed and analyzed as follows. The Pearson correlation coefficient between oocytes for replicates was >0.81 (**Figure 2A**). To allow the unsupervised analysis of the dataset, we performed PCA and transformed large gene expression data into a smaller and ideally more manageable dataset of composite variables. Then, the *Zp2^wt/wt^* (blue) and *Zp2^mut/mut^* (red) oocytes were clustered into two groups (**Figure 2B**).

To identify DEGs between the two groups, we selected genes with at least two-fold difference and an adjusted *P* value ≤ 0.05. Altogether, 474 DEGs were identified, of which 362 were upregulated and 112 were downregulated in the *Zp2^mut/mut^* oocyte (**Figure 2C**). This observation was supported by gene-based hierarchical clustering where the *Zp2^wt/wt^* and *Zp2^mut/mut^* oocytes were clustered into two major discrete trees (**Figure 2D**). These findings supported that there were fundamental molecular regulatory differences between the *Zp2^wt/wt^* and *Zp2^mut/mut^* oocytes.

To confirm the results of the scRNA-seq analysis, we analyzed the expression of four DEGs (two downregulated and two upregulated) by qRT-PCR. The results showed that the expressions of *Esrrg* and *Ehbp1* were significantly downregulated (*P* < 0.01), whereas those of *TP53* and *Eno1* were significantly upregulated (*P* < 0.05) (**Supplementary Figure 2**). This result is consistent with the scRNA-seq data.

### GO and KEGG analyses of DEGs

GO and KEGG enrichment analyses were performed to better understand the function of the DEGs. GO analysis was performed to highlight biological processes, molecular functions, and cellular components related to the DEGs (**Figure 2E**). This analysis showed many DEGs were involved in biological processes relevant to “transcription from RNA polymerase II promoter,” “negative regulation of cell proliferation,” and “regulation of immune response.” Cellular components involved were some organelles related to protein secretion and transport, such as “extracellular exosomes,” “Golgi bodies,” “endoplasmic reticulum,” and “extracellular matrix.” Molecular functions were mostly related to protein binding and DNA binding.

The KEGG analysis showed that the upregulated genes were enriched in 236 signaling pathways, including the tumor necrosis factor (TNF) signaling pathway, the Toll-like receptor signaling pathway, and cell adhesion molecules (CAMs). It also showed that the downregulated genes were enriched in 92 signaling pathways, including oxidative phosphorylation, TGF-β signaling, and cholesterol metabolism, which were closely related to oocyte growth and development (**Figures 2F, G**).

**Figure 3:**
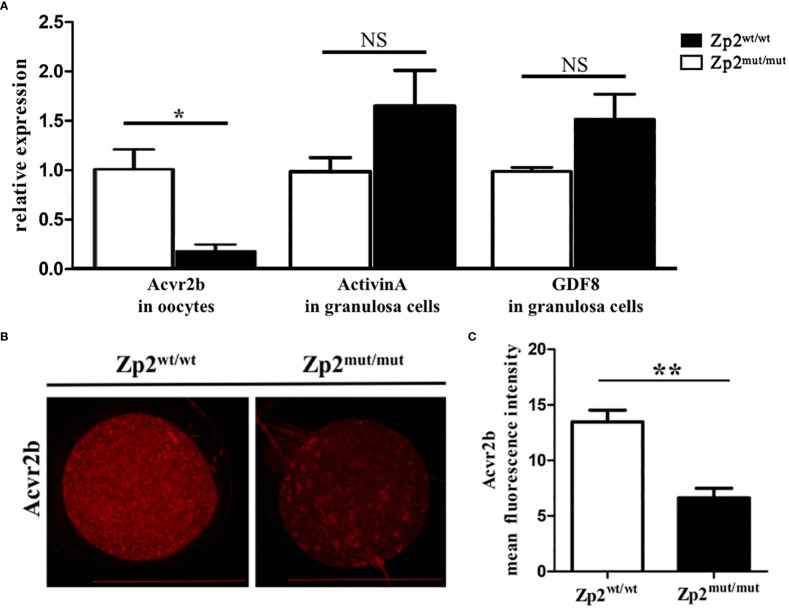
Expression levels of effector molecule in TGF-β signaling pathway. **(A)** The relative mRNA expression of Acvr2b (in oocytes, n=10) and ligand, Activin A and GDF8 (in granulosa cells, containing granulosa cells around 5 oocytes), was confirmed by RT-qPCR. Each treatment was repeated 3 times. *P < 0.05 represent significant differences between two groups. NS, not significant. **(B)** Immunofluorescence assay of Acvr2b for oocytes. Each treatment was repeated 3 times with each replicate containing about 5 oocytes. Scale bar: 100μm. **(C)** Mean fluorescence intensity of Acvr2b in two groups. **P < 0.01 represent significant differences between two groups.

DEGs enriched in the TGF-β signaling pathway and downstream effector molecules were further explored. *Acvr2b* mRNA levels in the *Zp2^mut/mut^* group were significantly lower than those in the *Zp2* ^wt/wt^ group (**Figure 3A**), consistent with the Acvr2b levels results using immunofluorescence assay (**Figures 3B, C**). Granulosa cells from the two groups of oocytes were collected to determine the mRNA levels of activin A and GDF8, the relevant ligands of Acvr2b, which showed no significant difference between the two groups (**Figure 3A**).

**Figure 4:**
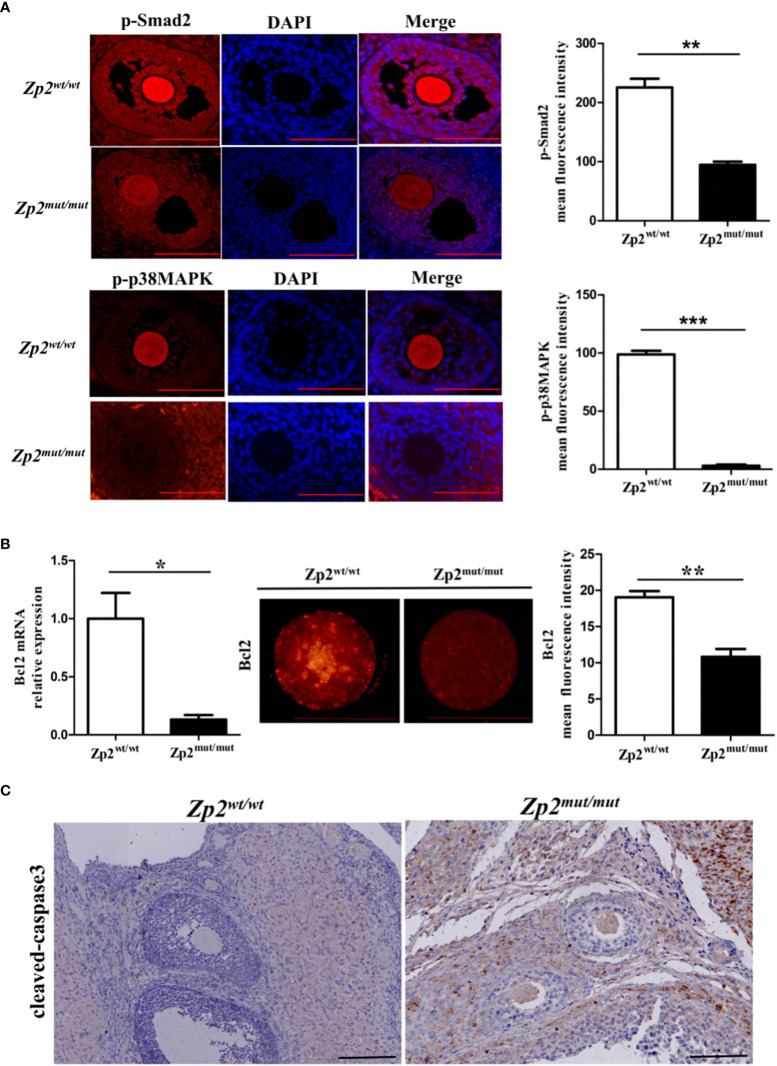
Phosphorylation level of Smad2 and p38MAPK in *Zp2^wt/wt^* and *Zp2^mut/mut^* rats. **(A)** Expression of p-smad2 and p-p38MAPK in ovary from *Zp2^wt/wt^* and *Zp2^mut/mut^* groups. (Left) Immunofluorescence assay of p-smad2 and p-p38MAPK for ovary. Each treatment was repeated 3 times. Scale bar: 100μm. (Right) Mean fluorescence intensity of p-Smad2 and p-p38MAPK in two groups. **P < 0.01 and ***P < 0.001 represent significant differences between two groups. **(B)** Expression of *Bcl2* in oocytes from *Zp2^wt/wt^* and *Zp2^mut/mut^* groups. (Left) The relative mRNA expression of *Bcl2* was confirmed by qPCR (n=10). (Center) Immunofluorescence assay of *Bcl2* for oocytes. Each treatment was repeated 3 times with each replicate containing about 5 oocytes. Scale bar: 100μm. (Right) Mean fluorescence intensity of *Bcl2* in two groups. NS: not significant. *P < 0.05 and **P < 0.01 represent significant differences between two groups. **(C)** Cleaved-caspase3 staining of ovaries in *Zp2^wt/wt^* and *Zp2^mut/mut^* groups. Each treatment was repeated 3 times.

The phosphorylation levels of Smad2 and p38MAPK were determined by performing an immunofluorescence assay of the ovary, which showed that their phosphorylation levels were significantly lower in the *Zp2^mut/mut^* group oocytes than in the *Zp2^wt/wt^* group oocytes (**Figure 4A**). The quantitative analysis of *Bcl2* by qRT-PCR and immunofluorescence assay showed that the mRNA and protein levels of *Bcl2* in the *Zp2^mut/mut^* group oocytes were significantly lower than those in the *Zp2^wt/wt^* oocytes (**Figure 4B**). Immunohistochemistry of the ovary showed many cleaved-caspase3 proteins in the *Zp2^mut/mut^* group oocytes, whereas cleaved-caspase3 protein was not found in the *Zp2^wt/wt^* group oocytes (**Figure 4C**).

## Discussion

In this study, we identified a homozygous nonsense mutation in *ZP2* (c.1924C>T, p.Arg642X) in an infertile woman with a thin or no ZP phenotype and total fertilization failure in routine IVF, which could be fertilized by ICSI. The abnormal form in the *ZP2* variant oocytes and less transferable embryos indicated an apparently detrimental effect of the *ZP2* variant.

Variants of *ZP2* has been reported to lead to abnormal ZP formation, recurrent IVF/ICSI failures and female infertility. Homozygous mutations accounted for the majority of *ZP2* variants. Oocytes retrieved from the patients with homozygous variants (c.1695-2A>G, c.1691_1694dup (11), c.1115G>C (8) and c.1235_1236del (10)) exhibited a thin or no ZP and was defective for sperm binding and penetration and led to IVF failure (11) or embryos arrest (8). One of them (c.1235_1236del) used ICSI for fertilization and could be fertilized and underwent cleavage successfully, but they failed to form a blastocyst (10). In addition, a compound- heterozygous mutation in *ZP2* gene (c.860_861del/c.1924C>T) was identified that oocytes had no ZP or an abnormal ZP with a thin matrix and an enlarged perivitelline space (7). Most recently, a heterozygous variant of *ZP2* has been identified to cause premature death of oocyte and EFS (12). In our study, a novel homozygous mutation in *ZP2* (c.1924C>T) was identified to lead to a thin or no ZP. The oocytes could be fertilized with ICSI, formed blastocysts, got implantation and delivered a healthy baby.

In addition to failure to fertilize, to explore the mechanism of ZP defects affecting the fertility of oocytes, we analyzed the transcriptomes of the *Zp2^wt/wt^* and *Zp2^mut/mut^* rat oocytes by scRNA-seq, and the expression of DEGs highlighted the organization of the extracellular matrix and signal communication between the oocytes and granulosa cells. The transcriptomes of the *Zp2^wt/wt^* and *Zp2^mut/mut^* rat oocytes were analyzed by scRNA-seq, and PCA divided them into two clusters. We further analyzed the “extracellular matrix” term and found 14 DEGs including *Dcn* and *Itgav*. The extracellular matrix is involved in cell migration, proliferation, growth, and development and plays an important role in mammalian ovarian functions and oogenesis. For example, some proteoglycans and collagens in the extracellular matrix of oocytes can participate in growth factor signaling during follicle development and provide rigid support for ovarian tissues (20–23). Itgav is an α integrin subunit and is considered a potential receptor for fibronectin 1 (FN1), which is a glycoprotein component of the extracellular matrix. A study has shown that FN1 is involved in early blastocyst formation (24). Decorin, a small leucine-rich proteoglycan, is a key regulator of extracellular matrix assembly. It can interact with TGF and epidermal growth factor receptors. Its aberrant expression is associated with oocyte maturation and oocyte quality (25, 26). Therefore, we speculate that ZP defects may affect the normal assembly of other extracellular matrices and adversely affect oocyte development.

The KEGG analysis of the DEGs showed upregulated pathways enriched in CAMs. The maintenance of follicle integrity mainly depended on adhesion molecules secreted by oocytes, such as cadherin and lectin. These adhesion molecules form point-like connections between oocytes and granulosa cells to support normal follicular morphology, and the granulosa cells are anchored around oocytes by adhesion connections (27, 28). The downregulated pathways were enriched in energy metabolism, including oxidative phosphorylation and cholesterol metabolism. The oxidative phosphorylation pathway is related to mitochondrial energy metabolism (29). Oocytes cannot utilize glucose efficiently as an energy source (30). Thus, granulosa cells metabolize glucose to pyruvate, which can then be transmitted *via* gap junctions to the oocytes, where it serves as an energy substrate (31). It is necessary for oocyte development (32, 33). This also explains why many DEGs were concentrated in oxidative phosphorylation pathways after ZP defects. In our study, the ZP was absent in some oocytes, and multiple granulosa cells were ectopic. It may be related to the maladjustment of cellular adhesion molecular pathways.

Besides, the downregulated pathways highlighted the TGF-β signaling pathway, which mediates oocyte survival and apoptosis (34). We verified that the key receptor Acvr2b in this pathway was significantly downregulated in the *Zp2^mut/mut^* rats, whereas the relevant ligands, such as activin A and GDF8, were not significantly different from those in the *Zp2^wt/wt^* rats. Activin A can promote the growth and development of follicles (35). Previous studies have shown that activin A is associated with oocyte maturation. Activin A can promote the cytoplasmic maturation of oocytes, enhance the developmental ability of oocytes, and increase the fertilization rate (36–38). A study confirmed that GDF8 can promote the cytoplasmic maturation of oocytes and enhance the meiotic capacity *via* the TGF-β signaling pathway (39). Hence, we confirmed that ZP defects affect the transport of activin A and GDF8 between oocytes and granulosa cells, which must have adverse effects on oocyte growth and development. This is consistent with our previous findings that ZP defects perturb normal signaling between oocytes and surrounding granulosa cells mediated by TZPs.

Smad2 and p38MAPK are the main downstream factors in the TGF-β signaling pathway, and these processes are essential for correct oocyte maturation before the ovulation stage (40). During the follow-up, we found that the phosphorylation levels of Smad2 and p38MAPK were significantly lower in the *Zp2^mut/mut^* group oocytes (ZP-absent), which indicated that the active signal of the TGF-β signaling pathway was weak for oocyte maturation. The Bcl2 family members are suggested to be one of the major players of apoptosis in the ovary (41, 42) and has been identified as a major proapoptotic regulator in controlling the mitochondrial apoptotic pathway (43). It has been suggested that interaction between the Fas system and Bcl2 family members may define the rate of apoptosis (42). Previous studies have shown that Bcl2 can be used as an upstream regulator of caspase3, which is a crucial terminal-cleaving enzyme involved in apoptosis (44–46). Compared with the wild-type oocytes, the decreased expression of Bcl2 and the activated caspase3 led to oocyte apoptosis in the *Zp2^mut/mut^* rats.

## Conclusions

In conclusion, our data based on human subjects and rat models showed the importance of ZP integrity for oocyte development. We are the first to investigate the effect of ZP defects on oocyte gene transcription. Our results confirmed that the disruption of the integrity of the ZP affects the signal communication between oocytes and granulosa cells and causes oocyte apoptosis, which would adversely affect the developmental potential of oocytes.

## Availability of data and materials

Original datasets are available in a publicly accessible repository: BioProject PRJNA947370.

## Data availability statement

The datasets presented in this study can be found in online repositories. The names of the repository/repositories and accession number(s) can be found in the article/**Supplementary Material**.

## Ethics statement

The studies involving human participants were reviewed and approved by the ethical review boards of the Second Xiangya Hospital, Central South University, China. The patients/participants provided their written informed consent to participate in this study. The animal study was reviewed and approved by The Animal Care and Use Committee, Central South University, China.

## Author contributions

JuZ, YS, JiZ, HX and H-LH conceived and designed the experiments. JiZ, XW, WS and JC enrolled the patients and collected clinical information. JuZ, YS, JiZ, RQ, DG and SW performed the experiments and analyzed the data. JuZ, YS and JiZ wrote the manuscript. JuZ, HX and H-LH edited the manuscript, with input from others. All authors read and approved the final manuscript.

## Supplementary material

Supplementary Figure 1 Images of oocytes for scRNA-seq. Scale bar: 100μm.

Supplementary Figure 2 Validation of scRNA-seq data of genes in oocytes from *Zp2^wt/wt^* and *Zp2^mut/mut^* groups. **(A)** Expression levels of Esrrg, Ehbp1, TP53 and Eno1 in scRNA-seq database. **(B)** The relative mRNA expression levels of selected genes were confirmed by RT-qPCR (n=10). Data are presented as the means ± SEM. **P* < 0.05 and ***P* < 0.01 represent significant differences between two groups.
