# Supplementary figures and images for: Identification of zona pellucida defects revealed a novel loss-of-function mutation in ZP2 in humans and rats
Source: Front Endocrinol (Lausanne). 2023 May 24;14:1169378. doi: 10.3389/fendo.2023.1169378 (PMC10244809; doi:10.3389/fendo.2023.1169378)

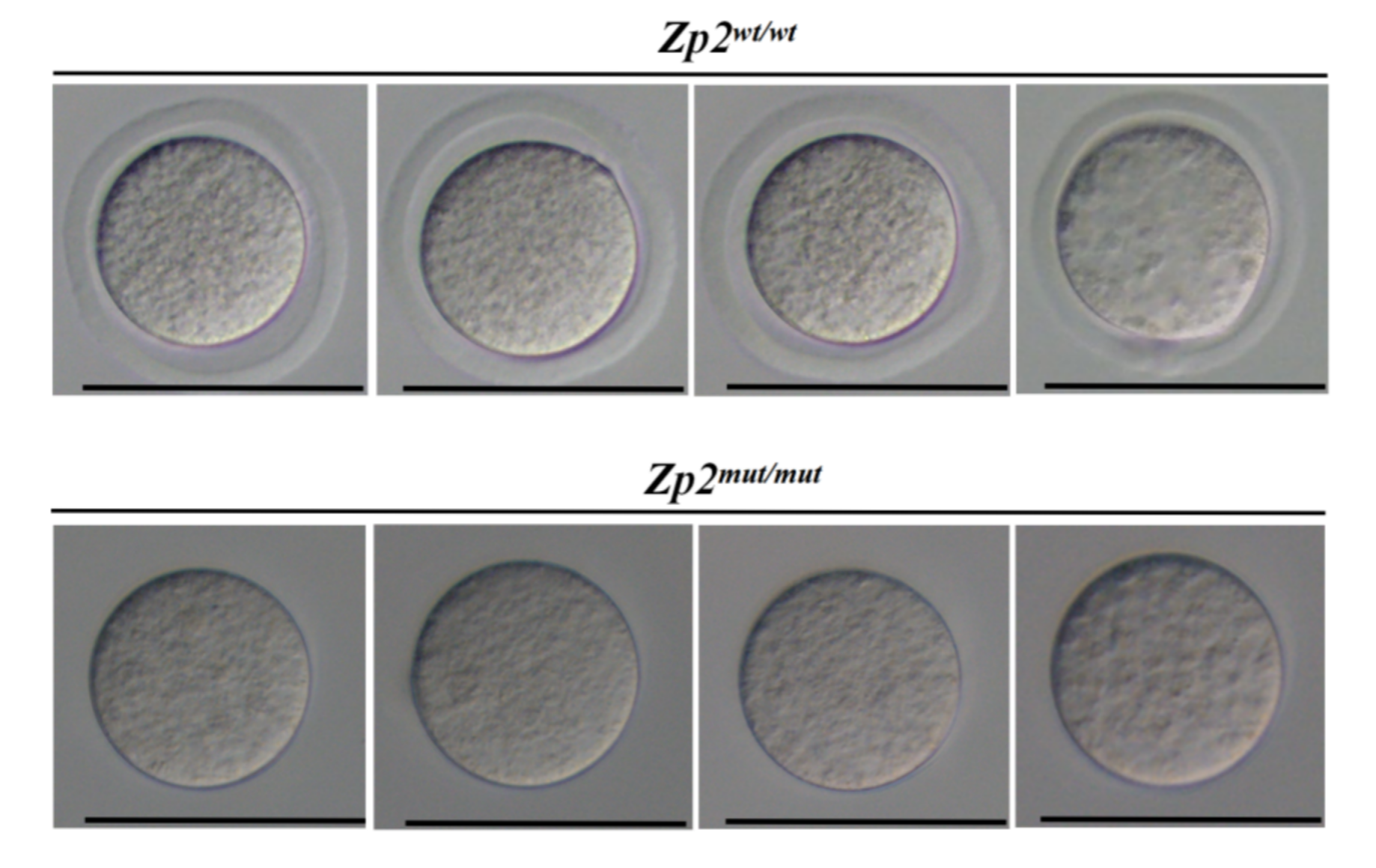

Supplement: Supplementary Figure 1 — Images of oocytes for scRNA-seq. Scale bar: 100μm. [file Image_1.tif]

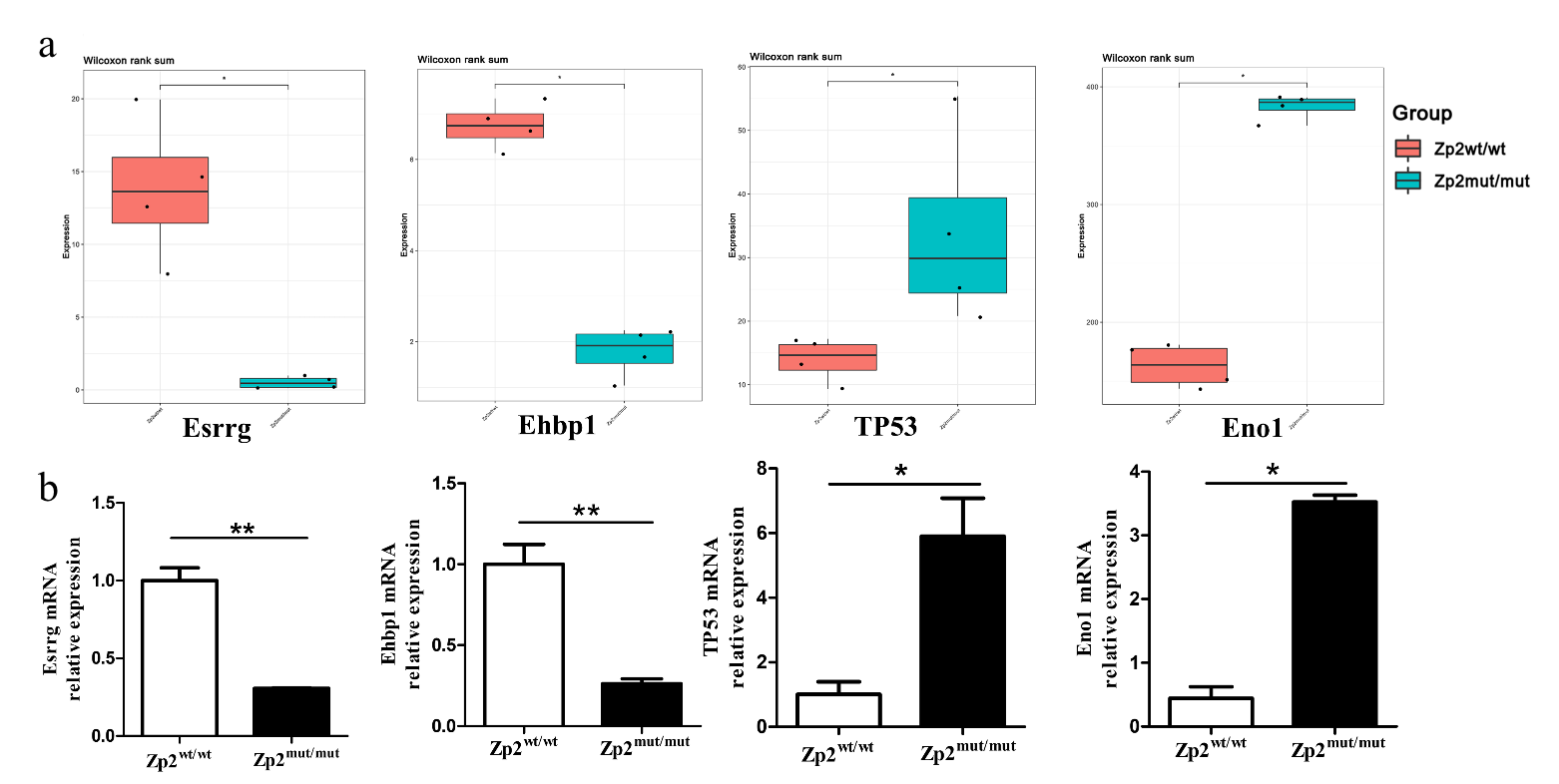

Supplement: Supplementary Figure 2 — Validation of scRNA-seq data of genes in oocytes from Zp2wt/wt and Zp2mut/mut groups. (A) Expression levels of Esrrg, Ehbp1, TP53 and Eno1 in scRNA-seq database. (B) The relative mRNA expression levels of selected genes were confirmed by RT-qPCR (n=10). Data are presented as the means ± SEM. *P < 0.05 and **P < 0.01 represent significant differences between two groups. [file Image_2.tif]
