# Supplementary material for: Identification of zona pellucida defects revealed a novel loss-of-function mutation in ZP2 in humans and rats
Source: Front Endocrinol (Lausanne). 2023 May 24;14:1169378. doi: 10.3389/fendo.2023.1169378 (PMC10244809; doi:10.3389/fendo.2023.1169378)
Supplement: Supplementary file 3 [file Table_1.docx]

**Supplementary Table 1** Primers for qRT-PCR.

| Primers | | Sequence (5'→3') | cDNA | Product size (bp) |
| --- | --- | --- | --- | --- |
| Pair 0 | Pair 1-F | ACCAAACTCTTGGAAGAGCCC | *Esrrg* | 131 |
|  | Pair 1-R | CTGCAGAGAAGCCTTTCCGA |  |  |
| Pair 1 | Pair 1-F | GTTGAGAACAGACCAGAGATGA | *Enbp1* | 133 |
|  | Pair 1-R | TGGTGTCTTTGAACCCTTTTTCA |  |  |
| Pair 2 | Pair 2-F | CCCCTGAAGACTGGATAACTGT | *Tp53* | 146 |
|  | Pair 2-R | ATTAGGTGACCCTGTCGCTG |  |  |
| Pair 3 | Pair 3-F | AGACTCCCTTCGGTGTCTCC | *Eno1* | 150 |
|  | Pair 3-R | TGCGGTGTAGAGATCCACCT |  |  |
| Pair 4 | Pair 4-F | TCTGGTGACCTCCTCGACCAA | *Acvr2b* | 91 |
|  | Pair 4-R | ATCCACTGAGTCTGGAGAAAGCC |  |  |
| Pair 5 | Pair 5-F | TCGCGACTTTGCAGAGATGT | *Bcl2* | 116 |
|  | Pair 5-R | CAATCCTCCCCCAGTTCACC |  |  |
| Pair 6 | Pair 6-F | TCATGCCAACTACTGCGAGG | *Activin A* | 124 |
|  | Pair 6-R | ACAGTGAGGACCCGGACG |  |  |
| Pair 7 | Pair 7-F | CAGACACACCCAAGAGGTCC | *GDF8* | 106 |
|  | Pair 7-R | AAGGCTTCGAAATCGACCGT |  |  |
| Pair 8 | Pair 8-F | CCCATCTATGAGGGTTACGC | *Beta-actin* | 159 |
|  | Pair 8-R | TTTAATGTCACGCACGATTTC |  |  |
