# Supplementary material for: Identification of zona pellucida defects revealed a novel loss-of-function mutation in ZP2 in humans and rats
Source: Front Endocrinol (Lausanne). 2023 May 24;14:1169378. doi: 10.3389/fendo.2023.1169378 (PMC10244809; doi:10.3389/fendo.2023.1169378)
Supplement: Supplementary file 4 [file Table_2.docx]

**Supplemental Table 2** Clinical features of the patient.

| Individual | Age (y) | Duration of infertility (y) | BMI | Basal FSH (IU/l) | Basal LH (IU/l) | Basal E_2_ (pg/ml) | Basal P (ng/ml) | Testo (ng/dl) | PRL (pg/ml) | AMH (ng/ml) |
| --- | --- | --- | --- | --- | --- | --- | --- | --- | --- | --- |
| II-3 | 30 | 7 | 21.3 | 4.69 | 3.18 | 49.67 | 0.15 | 80.12 | 10.29 | 1.48 |
| Reference limits |  |  |  | 3.03-8.08 | 2.4-12.6 | 12.4-233 | 0.057-0.893 | 2.33-59.48 | 5.18-26.53 | 0.67-10.92 |
